# Immature particles and capsid-free viral RNA produced by Yellow fever virus-infected cells stimulate plasmacytoid dendritic cells to secrete interferons

**DOI:** 10.1038/s41598-018-29235-7

**Published:** 2018-07-18

**Authors:** Laura Sinigaglia, Ségolène Gracias, Elodie Décembre, Matthieu Fritz, Daniela Bruni, Nikaïa Smith, Jean-Philippe Herbeuval, Annette Martin, Marlène Dreux, Frédéric Tangy, Nolwenn Jouvenet

**Affiliations:** 1 Viral Genomics and Vaccination Unit, UMR3569 CNRS, Institut Pasteur, Paris, France; 2 CIRI, Inserm U1111, CNRS UMR5308, École Normale Supérieure de Lyon, Université Claude Bernard Lyon 1, Lyon, France; 3 Molecular Genetics of RNA Viruses Unit, UMR3569 CNRS, Institut Pasteur, Paris, France; 4 Chemistry & Biology, Modeling & Immunology for Therapy, UMR8601 CNRS, Université Paris Descartes, Paris, France

## Abstract

Plasmacytoid dendritic cells (pDCs) are specialized in the production of interferons (IFNs) in response to viral infections. The *Flaviviridae* family comprises enveloped RNA viruses such as Hepatitis C virus (HCV) and Dengue virus (DENV). Cell-free *flaviviridae* virions poorly stimulate pDCs to produce IFN. By contrast, cells infected with HCV and DENV potently stimulate pDCs via short-range delivery of viral RNAs, which are either packaged within immature virions or secreted exosomes. We report that cells infected with Yellow fever virus (YFV), the prototypical flavivirus, stimulated pDCs to produce IFNs in a TLR7- and cell contact- dependent manner. Such stimulation was unaffected by the presence of YFV neutralizing antibodies. As reported for DENV, cells producing immature YFV particles were more potent at stimulating pDCs than cells releasing mature virions. Additionally, cells replicating a release-deficient YFV mutant or a YFV subgenomic RNA lacking structural protein-coding sequences participated in pDC stimulation. Thus, viral RNAs produced by YFV-infected cells reach pDCs *via* at least two mechanisms: within immature particles and as capsid-free RNAs. Our work highlights the ability of pDCs to respond to a variety of viral RNA-laden carriers generated from infected cells.

## Introduction

Plasmacytoid dendritic cells (pDCs) are rare immune cells that circulate in the blood where they represent on average 0.4% of the whole peripheral blood mononuclear cells (PBMCs)^1^. They migrate to peripheral lymphoid organs and peripheral tissues upon pathogen infection. They are specialized in the production of type I (mainly IFN-α and -β) and type III (IFN-λ) interferons (IFNs) in response to a variety of pathogens, including evolutionary distant viruses^1^. Secreted IFN-α/β and IFN-λs (IL-28a, IL-28b and IL-29) bind to their receptors and signal via the canonical Janus-activated kinase (Jak)–signal transducer and activator of transcription (STAT) pathway to trigger the expression of hundreds of antiviral IFN-stimulated genes^2^. Following internalization of circulating cell-free RNA viruses, pDCs are stimulated via recognition of viral ssRNA by the endosomal sensor TLR7^3^. Such sensing of viral nucleic acids occurs mainly independently of viral replication^4–7^. However, TLR7-mediated response can be coupled to viral replication when viral replication intermediates are delivered to TLR7-positive lysosomes by the process of autophagy^8^. Viral replication intermediates can also stimulate pDCs via recognition by the cytosolic sensor RIG-I, albeit not very efficiently^9^. In addition to cell-free viruses, pDCs encounter infected cells during viral infections. The IFN response to infected cells by pDCs is of higher magnitude than the one triggered by cell-free viruses and depends on cell-to-cell contacts, TLR7 signaling and viral replication in infected cells but not in pDCs^9–12^. Contact between infected cells and pDCs facilitate short-range delivery of immunostimulatory viral RNAs, which are either packaged within enveloped virions trapped at the site of cell-cell contacts, as described for retroviruses^13,14^, enveloped Hepatitis A virus^15^ or Dengue virus (DENV)^6^; or within secreted exosomes, as reported for Hepatitis C virus (HCV)^7^ and Lymphocytic Choriomeningitis Virus^16^.

The *Flaviviridae* family, which consists of the hepacivirus, flavivirus and pestivirus genera, includes numerous human and livestock pathogens^17^. The prototype member of the hepacivirus genus is the blood-borne hepatitis C virus (HCV). The flavivirus genus includes vector-borne disease agents, such as yellow fever virus (YFV), dengue virus (DENV), West Nile virus (WNV) and the emerging Zika virus. *Flaviviridae* are enveloped viruses harboring a single positive-strand RNA genome. The genome encodes a polyprotein that is cleaved into structural proteins, which constitute the virion (capsid (C), membrane precursor (prM) and envelope (Env)) and non-structural (NS) proteins, which coordinate RNA replication, viral assembly and modulate innate immune responses.

In humans, YFV primarily targets the liver, but other tissues, such as heart, kidneys and lungs, are also sites of replication^18^. Severe clinical symptoms include hemorrhagic fever and death. Proteomic-studies performed on PBMCs of subjects vaccinated with the attenuated YFV vaccine strain reported that transcripts coding for proteins involved in viral sensing and IFN signaling were up-regulated^19,20^. Moreover, recent mice studies showed that combined type-I and type-III IFNs are crucial for controlling YFV infection^21^. We previously showed that pDCs produced around 10 times less IFN-I when stimulated with cell-free YFV than with YFV-infected Vero cells^9^. However, the mechanisms by which YFV RNA are delivered from infected cells to pDCs remain to be elucidated. Here, we investigated these mechanisms using co-culture of YFV-infected hepatoma cells and primary human pDCs.

## Results

### YFV-infected Huh7.5 cells stimulate pDCs to produce IFN-α and IFN type-III via TLR7

**Figure 1:**
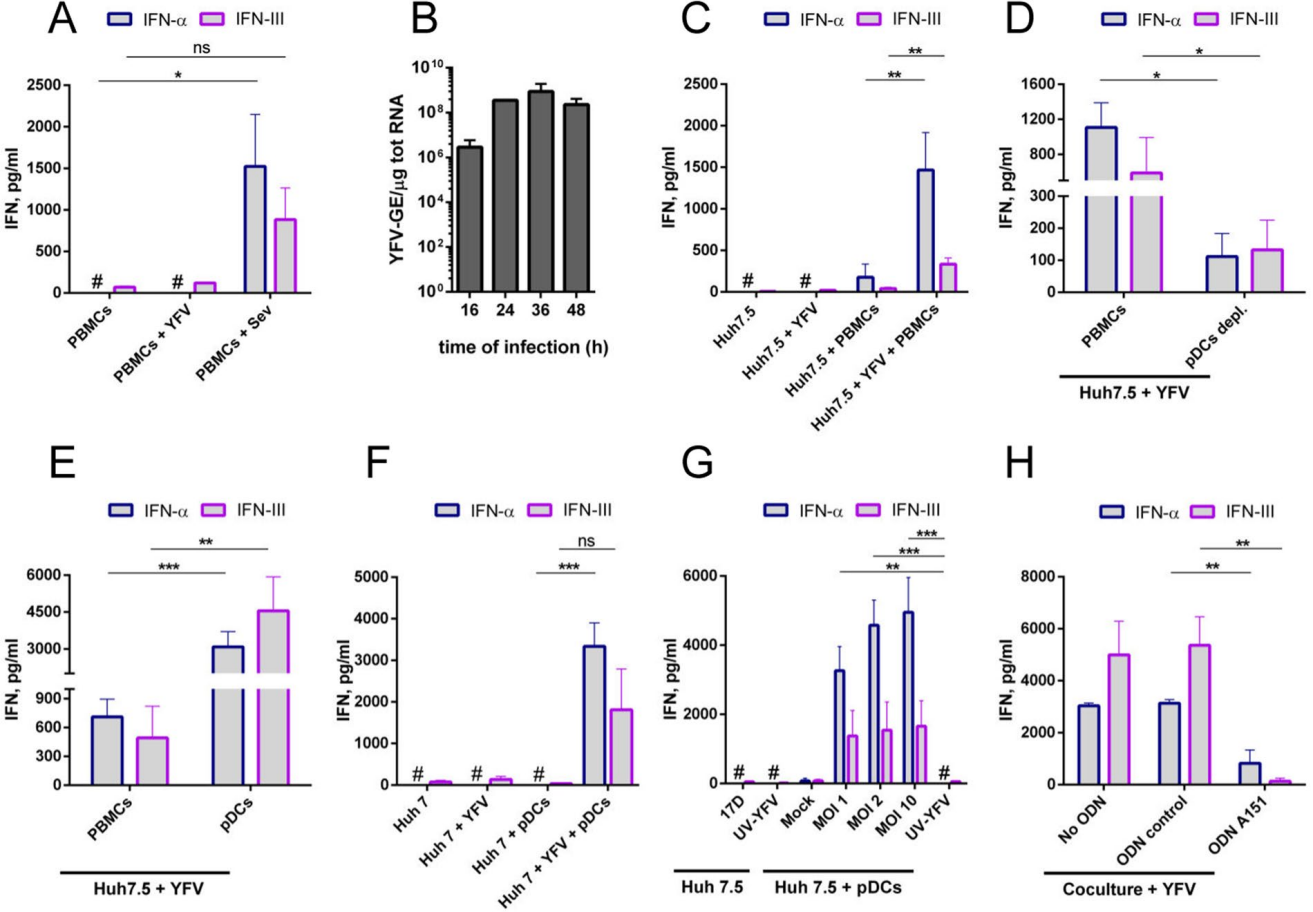
YFV-infected Huh7.5 cells stimulate pDCs to produce IFN-α and IFN type-III. YFV infections were carried out at a MOI of 1, unless stated otherwise. (**A,C–H**) Cell culture medium was analyzed by ELISA to determine the amount of IFN-α and IFN type-III secreted. ^#^Indicates values below the limit of detection of the assay. (**A**) Whole human PBMCs isolated from 5 blood donors were left uninfected or were infected with either cell-free YFV or Sendai virus (SeV) for 24 h. (**B**) Huh7.5 cells were infected with YFV for the indicated times (n = 3). The relative amounts of cell-associated viral RNA were determined by qPCR analysis and are expressed as genome equivalents (GE) per μg of total cellular RNA. (**C**) Huh7.5 cells were left uninfected or infected with YFV for 16 h. Alternatively, they were co-cultured with whole PBMCs for 24 h, or infected for 16 h with YFV and then co-cultured for a further 24 h with whole PBMCs. The experiment was performed with 5 blood donors. (**D**) Whole PBMCs or pDCs-depleted of PBMCs (‘pDCs depl.’) were co-cultured for 24 h with Huh7.5 cells infected with YFV for 16 h (n = 4). (**E**) Huh7.5 cells were infected for 16 h and then co-cultured for a further 24 h with whole PBMCs or purified pDCs (n = 4). (**F**) Huh7 cells were left uninfected or infected for 16 h. Alternatively, they were infected for 16 h and then co-cultured for a further 24 h with purified/enriched pDCs (n = 3). (**G**) Huh7.5 cells were infected with replication competent or UV-inactivated YFV for 16 h. Alternatively, Huh7.5 cells were left uninfected (mock) or infected for 16 h with different amounts of replication competent YFV or UV-inactivated YFV, and then co-cultured for a further 24 h with purified/enriched pDCs (n = 4). (**H**) pDCs were co-cultured for 24 h with 16 h-infected Huh7.5 cells in the presence or absence of a control ODN or the TLR7 inhibitor ODN A151 (n = 3). Error bars indicate the means ± SEM. Statistical analyses were performed using ANOVA (**A**,**C**,**F**,**G** and **H**) or student’s paired two-tailed t tests (**D** and **E**).

We examined whether PBMCs isolated from healthy donors produce IFNs in the presence of cell-free YFV virions. PBMCs were exposed for 24 hours to cell-free Sendai virus (SeV), a potent IFN inducer^22^, or to purified cell-free YFV (Fig. 1A). The attenuated strain YFV-17D was used since it replicates more efficiently in human cells than the parental strain Asibi^23^. Around 1500 pg/ml of IFN-α and 1000 pg/ml of IFN-III were secreted by PBMCs exposed to SeV (Fig. 1A). YFV-infected PBMCs failed to produce IFN-α and secreted as little IFN-III as non-stimulated cells (Fig. 1A). Huh7.5 hepatoma cells, which are extensively used in *Flaviviridae* research and physiologically relevant for YFV infection, were chosen to investigate whether PBMCs produced IFNs in the presence of YFV-infected cells. Huh7.5 cells were permissive to YFV, as shown by the levels of cell-associated viral transcripts detected by RT-qPCR at different times post-infection (Fig. 1B). Huh7.5 cells infected for 40 hours with YFV produced non-detectable levels of IFN-α and vey low levels of IFN-III (Fig. 1C). This was expected since Huh7.5 cells express a non-functional version of RIG-I^24^, which is a flavivirus RNA sensor^25^. Thus, the use of Huh7.5 cells as donor cells ensures that PBMCs represent the only source of IFN-α and the main source of IFN-III in the mixed cultures. PBMCs produced abundant IFN-α and -III when co-cultured for 24 hours with YFV-infected Huh7.5 cells (Fig. 1C). When isolated from PBMCs, pDC preparations typically showed a purity of more than 94%, as quantified by flow cytometry analysis (Fig. S1A). In pDC-depleted PBMCs cultured with infected Huh7.5 cells, IFN-α and -III production was significantly reduced compared to total PBMCs (Fig. 1D). Consistently, pDC-enriched population secreted significantly more IFN-α and IFN-III than PBMCs when co-cultured with infected Huh7.5 cells (Fig. 1E). Together, these data indicate that pDCs represent the main source of IFN-α and IFN type-III in human PBMCs co-cultured with YFV-infected Huh7.5 cells.

We assessed whether YFV-infected Huh7.5 cells were viable in the course of our experimental set-up, which comprises 16 hour-infection followed by 24 hours of co-culture with PBMCs or purified pDCs. Importantly, at these two time points, infected Huh7.5 cells were as viable as non-infected ones (Fig. S1B), suggesting that pDCs and PBMCs in the co-culture are mainly stimulated by viral genomes produced by infected Huh7.5 cells and not by danger signals generated by dying Huh7.5 cells.

We extended our analysis to the IFN-competent Huh7 parental cells. pDCs cultured with YFV-infected Huh7 cells produced around 3,000 pg/ml of IFN-α and 2,000 pg/ml of IFN type-III (Fig. 1F). Therefore, despite their difference in IFN competence, both Huh7 and Huh7.5 cells, once infected, are robust stimulators of pDCs. Huh7 cells produced around 100 pg/ml of IFN-III upon infection with cell-free YFV, which is around 3 times more that Huh7.5 cells (Fig. 1C,F). Therefore, to minimize pDC stimulation by IFN-III released from infected cells, co-culture experiments were performed with Huh7.5 cells.

We then compared the ability of Huh7.5 cells infected with UV-inactivated YFV or with different amounts of replicating-competent YFV to stimulate pDCs (Fig. 1G). pDCs co-cultured with Huh7.5 cells exposed to UV-inactivated YFV did not produce IFN-α and secreted as little IFN-III as non-infected cells, suggesting that viral replication in Huh7.5 cells is required for pDC stimulation. The relationship between amounts of infectious viruses used to infect Huh7.5 cells and secreted amounts of IFN by pDCs was not linear (Fig. 1G), suggesting that there is plateau for IFN production by pDCs.

TLR7 was previously shown to be involved in IFN production by pDCs sensing of cells infected with a variety of RNA viruses^6,10,12–15^. To evaluate the contribution of TLR7 in IFN secretion by pDCs exposed to infected Huh7.5 cells, pDCs were incubated with A151, an oligonucleotide inhibitor of TLR7^4,5^ or with a control oligonucleotide. Production of IFN-α and IFN-type-III was significantly reduced in pDCs treated with A151 as compared to cells treated with the control oligonucleotide upon co-culture with infected Huh7.5 cells (Fig. 1H). These experiments identified TLR7 as the main sensor of viral RNA in pDCs co-cultured with YFV-infected Huh7.5 cells.

### Physical contact between YFV-infected Huh7.5 cells and pDCs is required for IFN production

**Figure 2:**
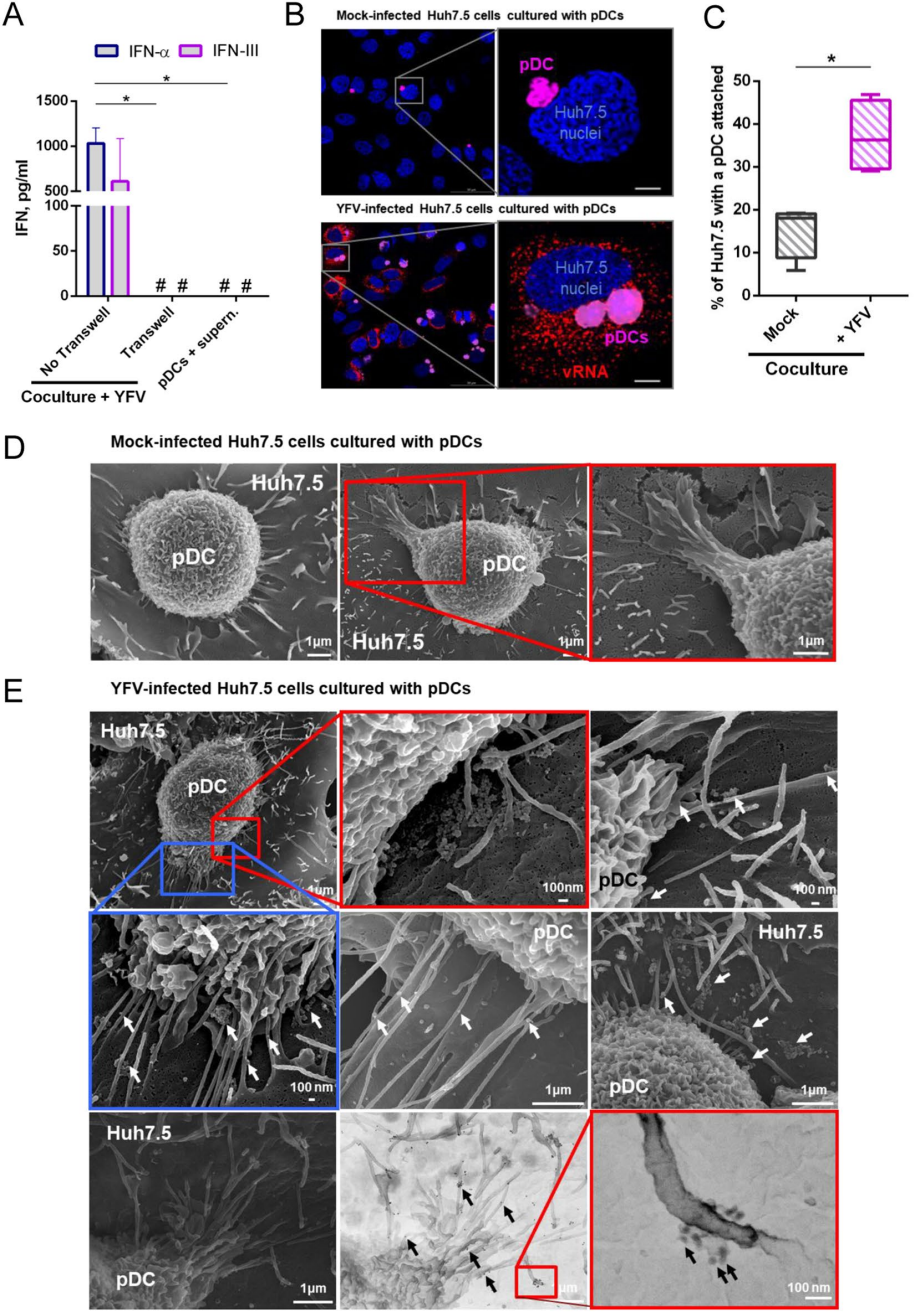
Contact between YFV-infected Huh7.5 cells and pDCs is required for IFN production. YFV infections were carried out at a MOI of 1. (**A**) Infected Huh7.5 cells and pDCs were co-cultured in wells in which they were separated, or not, by a transwell chamber. Alternatively, pDCs were incubated with supernatant collected from 16 h-infected Huh7.5 cells. Cell culture medium was analyzed by ELISA to determine the amounts of IFN-α and IFN-III secreted by cells. Data are means ± SEM of three independent experiments. Statistical analysis was performed using an ANOVA test. (**B**) Huh7.5 cells were infected with YFV for 16 h and then co-cultured with deep red-labeled pDCs (magenta) for a further 24 h. Cells were stained with NucBlue to visualize nuclei (blue) and then processed for FISH using a probe specific for vRNA (red). Images are representative of four independent experiments. Five microscopic fields were analyzed per experiment. Scale bars are 5 μm. (**C**) Quantification of the percentage of Huh7.5 cells associated with at least one pDC from the experiments represented in panel B. The line inside the boxes shows the mean values and the whiskers represent the minimal and maximal values of three independent experiments. Statistical analysis was performed using a paired t-test. (**D,E**) SEM analysis of the contact between pDCs and mock-infected or YFV-infected Huh7.5 cells. Images are representative of three independent experiments, in which at least twenty images per condition were taken. Examples of pDCs bound to mock-infected (**D**) or YFV-infected Huh7.5 cells (**E**) 24 h post co-culture are shown. (**E**) White arrows show objects resembling viruses. The bottom panels show the same view visualized using the secondary electron (SEI, left panel) or black scatter electron (BSE) detectors to observe cell morphology and the presence of colloidal gold staining Env as small black dots (black arrows). The 2 images were superimposed and reported as merged images (middle panel). In all panels, enlarged views of the images are indicated with a red or blue square. Scale bars are as indicated.

We investigated whether contact between infected Huh7.5 cells and pDCs was required to trigger IFN secretion. Production of both IFN-α and IFN-III was completely abrogated when infected Huh7.5 cells and pDCs were separated by a permeable transwell chamber (Fig. 2A). When pDCs were exposed to crude cell-free supernatant harvested from the same number of infected Huh7.5 cells than in the co-culture experiments, they did not produce IFN-α or type-III (Fig. 2A). These data revealed that physical contact between infected cells and pDCs is necessary for IFN production. The results also indicate that non-concentrated viral materials released from Huh7.5 cells, such as cell-free virions, are not able to stimulate pDCs.

We then investigated whether YFV infection promotes contact between pDCs and Huh7.5 cells. Infected Huh7.5 cells were identified by the presence of viral RNA (vRNA), which was detected using RNA fluorescence *in situ* hybridization (FISH) approach coupled with immunofluorescence and confocal microscopy. The FISH probe produced no signal in non-infected control Huh7.5 cells (Fig. 2B, upper panels) and a bright punctate signal in the cytoplasm of infected cells (Fig. 2B, lower panels), confirming the specificity of the probe. The analysis revealed that around 15% of non-infected Huh7.5 cells were in contact with at least one pDC (Fig. 2B,C). When infected for 40 hours, significantly more Huh7.5 cells (around 35%) were in contact with at least one pDC (Fig. 2B,C). Of note, by contrast with non-infected cells, the majority of infected Huh7.5 cells exhibited contacts with more than one pDC (Fig. 2B). These data suggest that YFV infection stimulates and/or prolongs contacts between the two cell types.

Scanning electron microscopy (SEM) analyses revealed that pDCs were connected to Huh7.5 cells, even in the absence of the virus (Fig. 2D), confirming confocal analysis (Fig. 2B, upper panels). Filopodia-like structures, which might originate from both cell types, were observed between pDCs and Huh7.5 cells, infected or not (Fig. 2D,E). These filopodia-like connections were present at all cell-cell contacts. Objects reminiscent of viruses, sometimes associated in clusters, were observed in the vicinity of, or on, these cell-cell connections (Fig. 2E, middle panels, white arrows). Immunogold labeling showed that these viral-like particles were positive for YFV Env (Fig. 2E, bottom panels, black arrows and Fig. S2). Since tight contact with infected cells is required for the stimulation of pDCs (Fig. 2A), these connections might provide the channel through which viral material is transferred from infected cells to pDCs.

### pDCs stimulated by infected Huh7.5 cells are positive for YFV RNA

**Figure 3:**
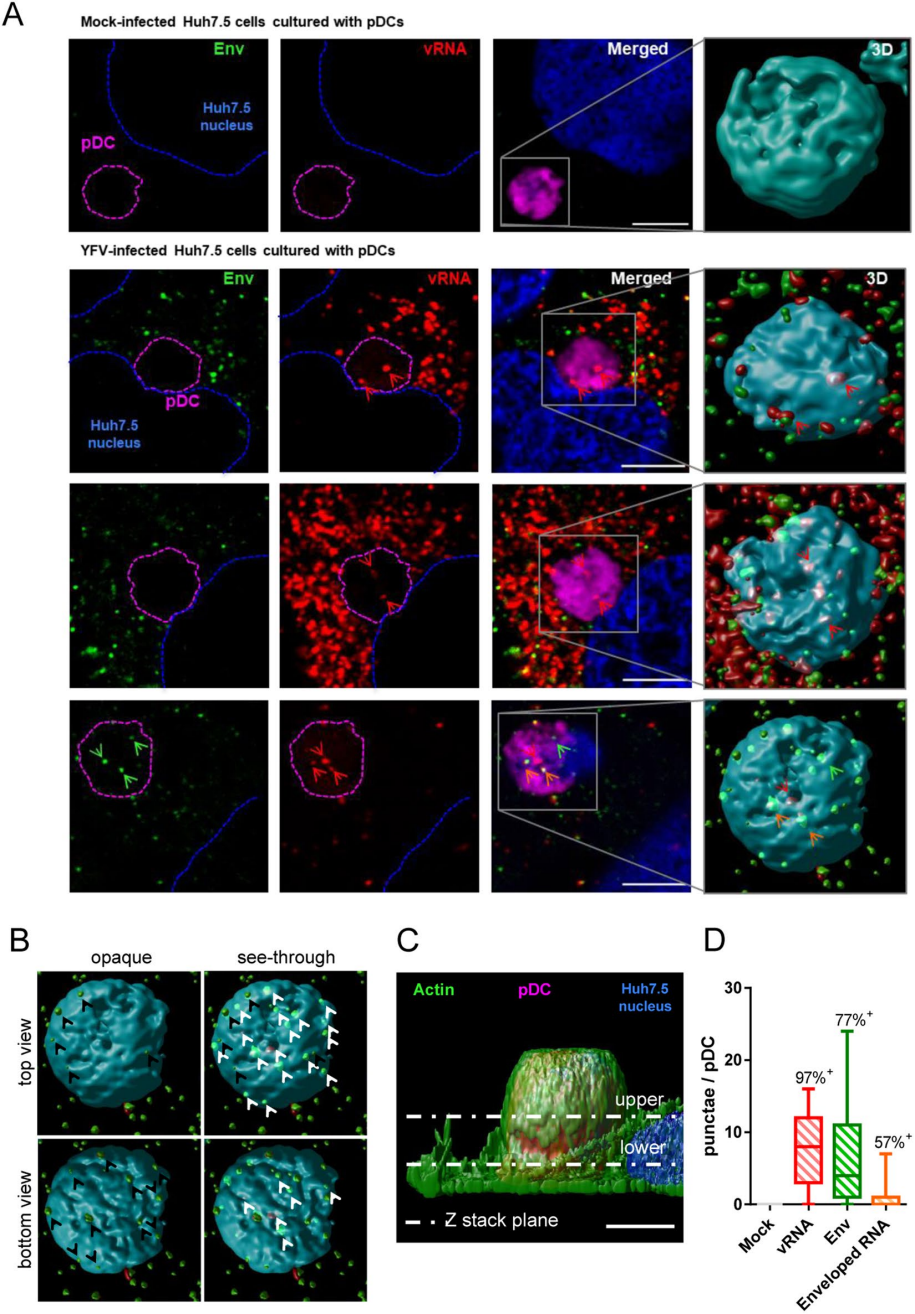
pDCs in close proximity of infected Huh7.5 cells are positive for YFV RNA. YFV infections were carried out at a MOI of 1. (**A**) Huh7.5 cells were left uninfected (mock) or were infected for 16 h and then co-cultured with deep-red-labeled pDCs (magenta) for a further 24 h. Cells were stained with antibodies specific for viral Env protein (green) and with NucBlue to visualize nuclei (blue). Cells were then processed for FISH using a probe specific for YFV (+) strand RNA (red). Blue dotted lines outline the boundaries of Huh7.5 nuclei. Magenta dotted lines represent pDC contours. Right images are enlarged 3D representation of the indicated pDCs. Red and green arrowheads indicate vRNA and Env proteins, respectively. Orange arrowheads show association of vRNA and Env protein signals. Images are representative of three independent experiments. Five microscopic fields were analyzed per experiment. Scale bars are 5 μm. (**B**) Comparison of opaque with see-through rendering of a 3D representation of top and bottom view of the pDC shown in the bottom right panel of A. Dots that appeared in the opaque view depict molecules that are on the surface of pDCs (black arrowheads), while see-through view revealed signals that are inside pDCs (white arrowheads). (**C**) 3D view of a Z-stack image of a Huh7.5 cell co-cultured with a deep-red-labeled pDC (magenta) and stained with anti-actin antibodies (green) and NucBlue to visualize nuclei (blue). The dotted lines are examples of single Z-stack planes. Scale bar is 5 μm. (**D**) Quantification of the number of dots representing vRNA, viral Env or vRNA associated with viral Env (enveloped RNA) detected in the 3D see-through view of individual pDC, as shown in B. The percentage of pDC containing at least one dot is reported on top of each box. The line inside the boxes shows the mean values and the whiskers represent the minimal and maximal values of three independent experiments in which thirty microscopic fields were analyzed.

vRNAs are natural ligands for TLR7^26,27^. FISH approach coupled with immunofluorescence and confocal microscopy was used to detect the presence of vRNA in the cytoplasm of pDCs that were in close proximity of infected Huh7.5 cells (Fig. 3A). Z-sections that crossed pDC full-volume were acquired and 3D rendering of these sections were generated to enable a comprehensive localization of vRNA and Env signals (Fig. 3A). Both vRNA and Env seemed to be associated with pDCs in contact with infected Huh7.5 cells (Fig. 3A, red and green arrowheads, respectively). Some vRNA molecules were associated with viral Env (Fig. 3A, bottom panels, orange arrowheads). Comparison of opaque with see-through rendering of the 3D images was used to determine whether vRNA and Env molecules were inside pDCs (Fig. 3B). Signals that appeared in the see-through rendering, but not in the top- or bottom-views of the opaque rendering, were considered internalized by pDCs (Fig. 3B, bottom right panel, white arrowheads). Env signals observed around pDCs represent virions in the cytoplasm of Huh7.5 cells, which are underneath pDCs, as depicted in Fig. 3C. Quantification analysis based on comparison between opaque and see-through renderings of the 3D images revealed that the vast majority (around 97%) of pDCs that were in close proximity of infected Huh7.5 cells contained at least one molecule of vRNA (Fig. 3D). On average, 8 molecules of vRNA were detected in the cytoplasm of these cells. 77% of pDCs in contact with infected cells were positive for the viral Env (Fig. 3D). Around 1 in 8 vRNA molecules were associated with viral Env, suggesting that the majority of vRNAs present in pDC cytoplasm were at a post-fusion stage at the time of the analysis. Alternatively, vRNA may be internalized in pDCs without being associated with viral envelope.

**Figure 4:**
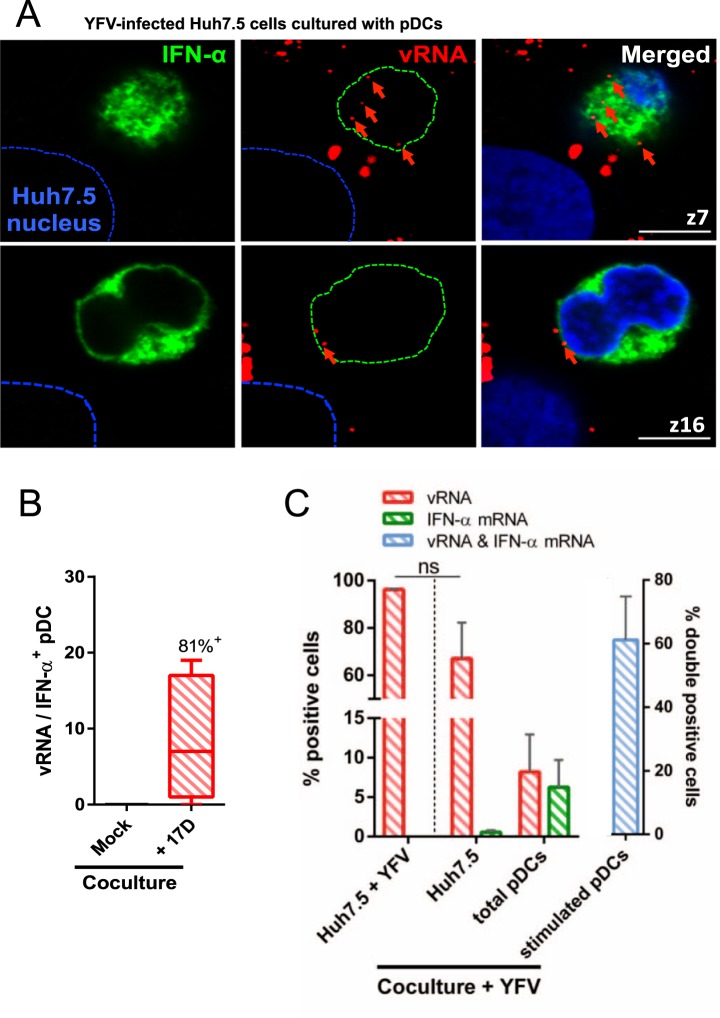
pDCs in close proximity of infected Huh7.5 cells are stimulated. YFV infections were carried out at a MOI of 1. Huh7.5 cells were infected for 16 h and then co-cultured with pDCs for a further 24 h. (**A**) Cells were stained with antibodies specific for all IFN-α subtypes, at the exception of IFN-α2b (green) and with NucBlue to visualize nuclei (blue). Cells were then processed for FISH using a probe specific for YFV (+) strand RNA (red). Two Z-stacks of the same image are shown. Images are representative of three independent experiments. Ten microscopic fields were analyzed. Scale bars are 5 μm. (**B**) Quantification of the number of vRNA punctae within the cytoplasm of IFN-α positive pDCs. The line inside the boxes shows the mean values and the whiskers represent the minimal and maximal values of three independent experiments. Ten microscopic fields were analyzed. The percentage of IFN-α positive pDC containing at least one vRNA puncta is reported on top of the bar. (**C**) Huh7.5 cells were infected for 40 h or, after 16 h, were co-cultured with eFluor 450-labeled pDCs for a further 24 h. Cells were incubated with probes that target vRNA and IFN-α1/2 mRNA, and then analyzed by flow cytometry. Representative flow panels are shown in Fig. S3. Data are means ± SEM of three independent experiments. Statistical analysis was performed using a paired t-test.

To determine whether pDCs that were positive for vRNA were stimulated, infected Huh7.5 cells co-cultured with pDCs were processed for FISH and stained with antibodies recognizing all IFN-α subtypes, at the exception of IFN-α2b (Fig. 4A). Quantification analysis revealed that 81% of IFN-α-positive pDCs in contact with infected Huh7.5 cells contained at least one molecule of viral RNA (Fig. 4A,B). pDCs positive for IFN-α but negative for vRNA may be stimulated in a autocrine manner by IFN-α or IFN type III. Of note, pDCs positive for IFN-α were often multinucleated (Fig. 4A). We have previously observed similar nuclear morphological changes in pDCs stimulated by cell-free Human Immunodeficiency Virus 1 (HIV-1)^28^. Our microscopic analysis was performed only on pDCs that were bound to Huh7.5 cells. To determine the proportion of stimulated cells within the whole pDC population (pDCs attached to Huh7.5 cells and pDCs in suspension), FISH coupled with flow cytometry (Flow-FISH) was performed^29^. We first assessed the specificity of the probes targeting vRNA and mRNA of human IFN-α subtypes 1 and 2 by Flow-FISH assays in Huh7.5 cells infected for 24 hours with YFV. More than 90% of infected Huh7.5 cells were positive for vRNA (Figs 4C and S3). Infected Huh7.5 cells were negative for IFN-α mRNA (Figs 4C and S3), confirming IFN-α ELISA analysis (Fig. 1C). When co-cultured with pDCs, around 70% of infected Huh7.5 cells were positive for vRNA (Figs 4C and S3), which is less than in the absence of pDCs. Albeit this difference is not statistically significant, it could be explained by the antiviral activity of the IFNs produced by pDCs. When mixed with infected Huh7.5 cells, around 7% of total pDCs were positive for vRNA and 6% for IFN-α mRNA (Figs 4C and S3), indicating that only a small fraction of pDCs were stimulated. Around 60% of stimulated pDCs were positive for vRNA (Figs 4C and S3). Again, pDCs positive for IFN-α but negative for vRNA might have been stimulated in an autocrine manner by IFNs. Together, these experiments indicate that the vast majority of pDCs connected to infected cells contained at least one molecule of vRNA, whereas a minority of the total number of pDCs contained vRNA, confirming that cell-cell contact greatly facilitate intercellular transfer of immunostimulatory RNAs.

### Infectious viruses released by infected Huh7.5 cells do not participate significantly in PBMCs stimulation

**Figure 5:**
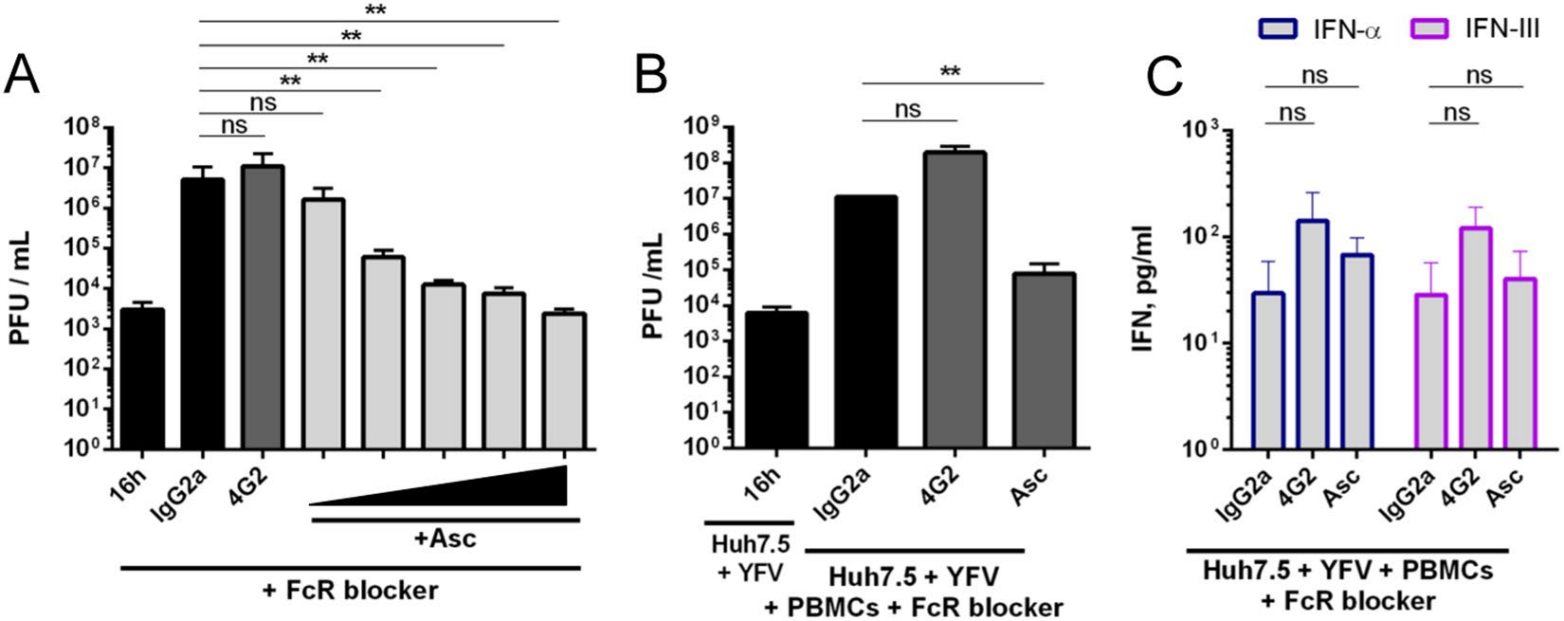
Infected Huh7.5 cells stimulate PBMCs in the presence of anti-YFV ascites fluid. YFV infections were carried out at a MOI of 0,2. (**A**) Huh7.5 cells were infected for 40 h, unless stated otherwise, in the absence or presence of the indicated antibodies. Presence of infectious viruses in the cell culture medium was assessed by plaque assay and expressed as PFU/ml (n = 4). (**B**) Huh7.5 cells were infected for 16 h. Alternatively, they were infected for 16 h and then co-cultured with whole PBMCs (treated with FcR blocking reagent) for a further 24 h, in the presence or absence of the indicated antibodies. Presence of infectious viruses in the cell culture medium was assessed by plaque assay and expressed as PFU/ml (n = 4) (**C**) Infected Huh7.5 cells were co-cultured with FcR blocking reagent-treated whole PBMCs exactly as in panel B. The amounts of IFN-α and IFN-III in the cell culture medium were assessed by ELISA (n = 3). Data in A to C are means ± SEM. Statistical analyses were performed using an ANOVA test.

To assess the potential role of released virions in pDC stimulation, infected Huh7.5 cells cultured with PBMCs were treated with anti-YFV polyclonal ascites fluid. These antibodies are potent inhibitors of YFV Env mediated events^23^, probably by neutralizing viral attachment to cell membranes and/or fusion between viral and endosomal membranes^30^. Consistent with our previous data^23^, the addition of increasing concentrations of anti-YFV ascites fluid in the supernatant of infected Huh7.5 cells from 16 to 40 hours post-infection resulted in a dose-dependent decrease in infectious viral titers (up to 4 log) compared to the addition of 4G2, an antibody that binds to viral Env but is unable to neutralize cell-free virions (Fig. 5A). Such a decrease in virus production suggests that anti-YFV ascites fluid neutralize all infectious particles released during this time window, which corresponds to the co-culture. When infected Huh7.5 cells were co-cultured with PBMCs in the presence of ascites fluid and Fc receptor (FcR) blocking antibodies, to avoid immune complexes internalization by pDCs and subsequent stimulation, 2 log less infectious particles were detected in cell culture media, compared to IgG2A-treated culture (Fig. 5B). ELISA revealed that the presence of the neutralizing anti-YFV ascites fluid did not reduce IFN-α and IFN-III production by PBMCs cultured with infected Huh7.5 cells (Fig. 5C), suggesting that infectious viruses released by infected Huh7.5 cells did not participate significantly in PBMC stimulation. Virions or viral RNA might traffic from infected Huh7.5 cells to pDCs without being released in the extra-cellular milieu, for instance within the filopodia-like connections observed by SEM. Alternatively, viral RNA might be delivered to pDCs by a mechanism that is resistant to neutralizing antibodies, such as, for instance, *via* packaging within non-infectious immature particles.

### Immature YFV particles produced by infected Huh7.5 cells stimulate pDCs

**Figure 6:**
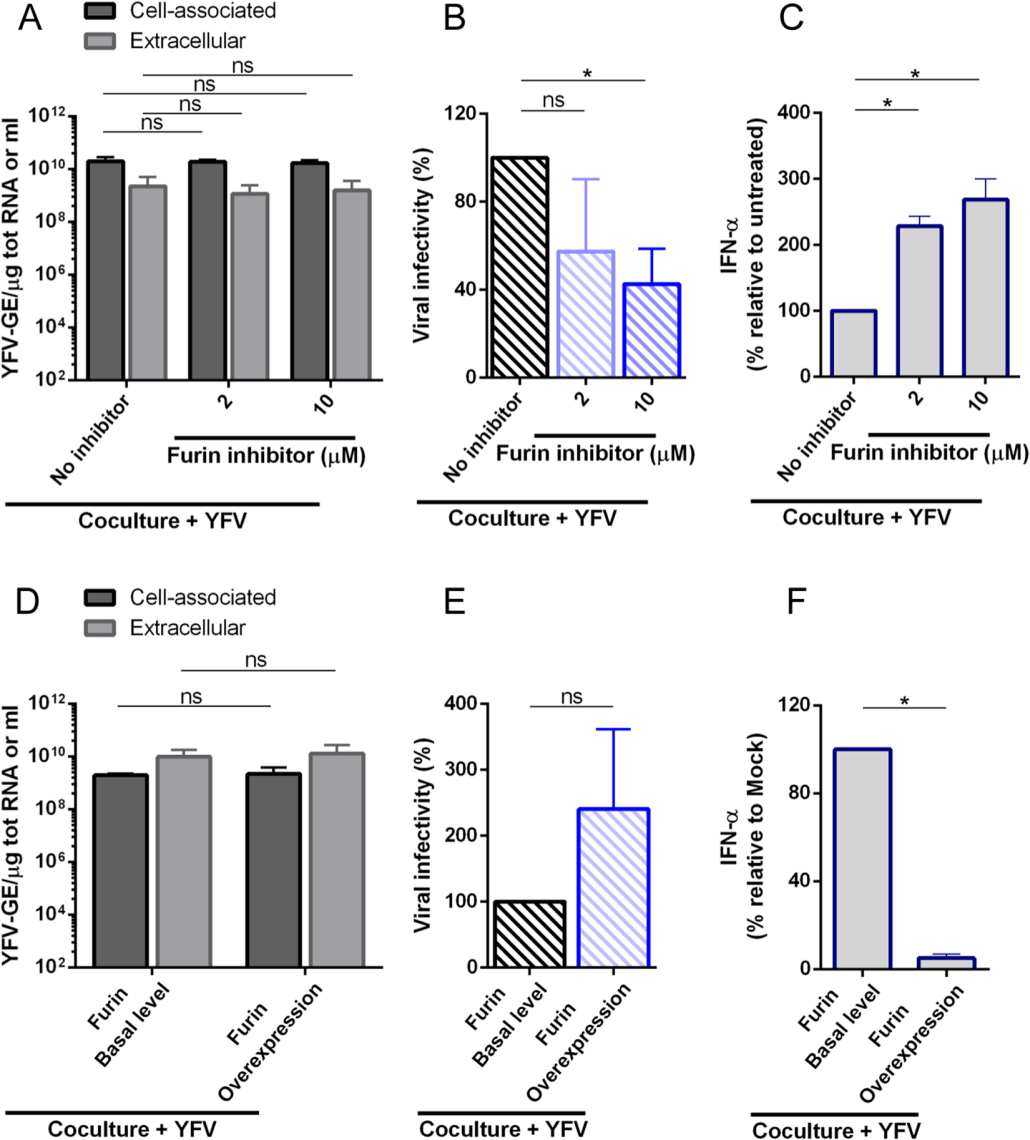
Immature YFV particles produced by infected Huh7.5 cells are robust stimulator of pDCs. YFV infections were carried out at a MOI of 1. (**A–C**). Huh7.5 cells were infected for 16 h and treated, or not, with the indicated concentration of furin inhibitor. They were then co-cultured with pDCs for a further 24 h. (**A**) The relative amounts of cell-associated and extracellular viral RNA were determined by qPCR analysis. Results are expressed as genome equivalents (GE) per μg of total cellular RNA or per ml (n = 3). (**B**) Presence of infectious viruses in media of infected cells treated or not with furin inhibitor was assessed by plaque assay. PFU per ml (means ± SD) of three independent experiments were expressed as percentage relative to non-treated cells. (**C**) Cell culture medium was analyzed by ELISA to determine the amount of secreted IFN-α. The results of three independent experiments were expressed as percentage relative to non-treated cells. (**D–F**) 293T cells, wild-type or stably over-expressing furin, were infected for 16 h with YFV and then co-cultured with pDCs for a further 24 h. (**D**) The relative amounts of cell-associated and extracellular viral RNA were determined by qPCR analysis. Results are expressed as genome equivalents (GE) per μg of total cellular RNA or per ml (n = 3). (**E**) Presence of infectious viruses in media of infected cells was assessed by plaque assay. PFU per ml (means ± SD) of three independent experiments were expressed as percentage relative to wild-type cells. (**F**) Cell culture medium was analyzed by ELISA to determine the amount of secreted IFN-α. The results of three independent experiments were expressed as percentage relative to wild-type cells. Statistical analyses were performed using ANOVA (**A**,**B** and **C**) or student’s paired two-tailed t tests (**D**,**E** and **F**).

Flavivirus-infected cells secrete a mixture of mature, partially immature, and fully immature particles into the extracellular space. Mature virions are highly infectious, whereas immature virions are not, largely because inefficiently cleaved prM proteins inhibits cell attachment and fusion of the virus^31^. To investigate the potential immunostimulatory activities of immature particles, pDCs were co-cultured with infected Huh7.5 cells treated with an inhibitor of furin, a Golgi-resident protease responsible for the cleavage of prM proteins. The levels of intra- and extra-cellular YFV RNA were not affected by the furin inhibitor (Fig. 6A). YFV-infected Huh7.5 cells treated with 10 μM of furin inhibitor produced significantly less infectious particles than control cells (Fig. 6B). pDCs produced significantly more IFNα when co-cultured with infected Huh7.5 cells treated with furin inhibitor than when co-cultured with non-treated cells (Fig. 6C). These results suggest that activation of pDCs triggered by contact with infected cells inversely correlates with the levels of prM maturation. We then took advantage of a previously described 293T cell line stably over-expressing furin^6^. These cells were infected with YFV and then co-cultured with pDCs. The levels of intracellular and extracellular YFV RNA were unaffected by furin over-expression (Fig. 6D). Cells over-expressing furin produced around 2 to 3 fold more infectious YFV particles than control cells (Fig. 6E). Cells producing more mature particles than control cells were impaired at stimulating co-cultured pDCs (Fig. 6F). Together, these data suggest that cells producing immature YFV particles are more potent at inducing IFNα production by pDCs than cells releasing mature virions.

### Env- and capsid-free viral RNA produced by infected cells participates in pDC stimulation

**Figure 7:**
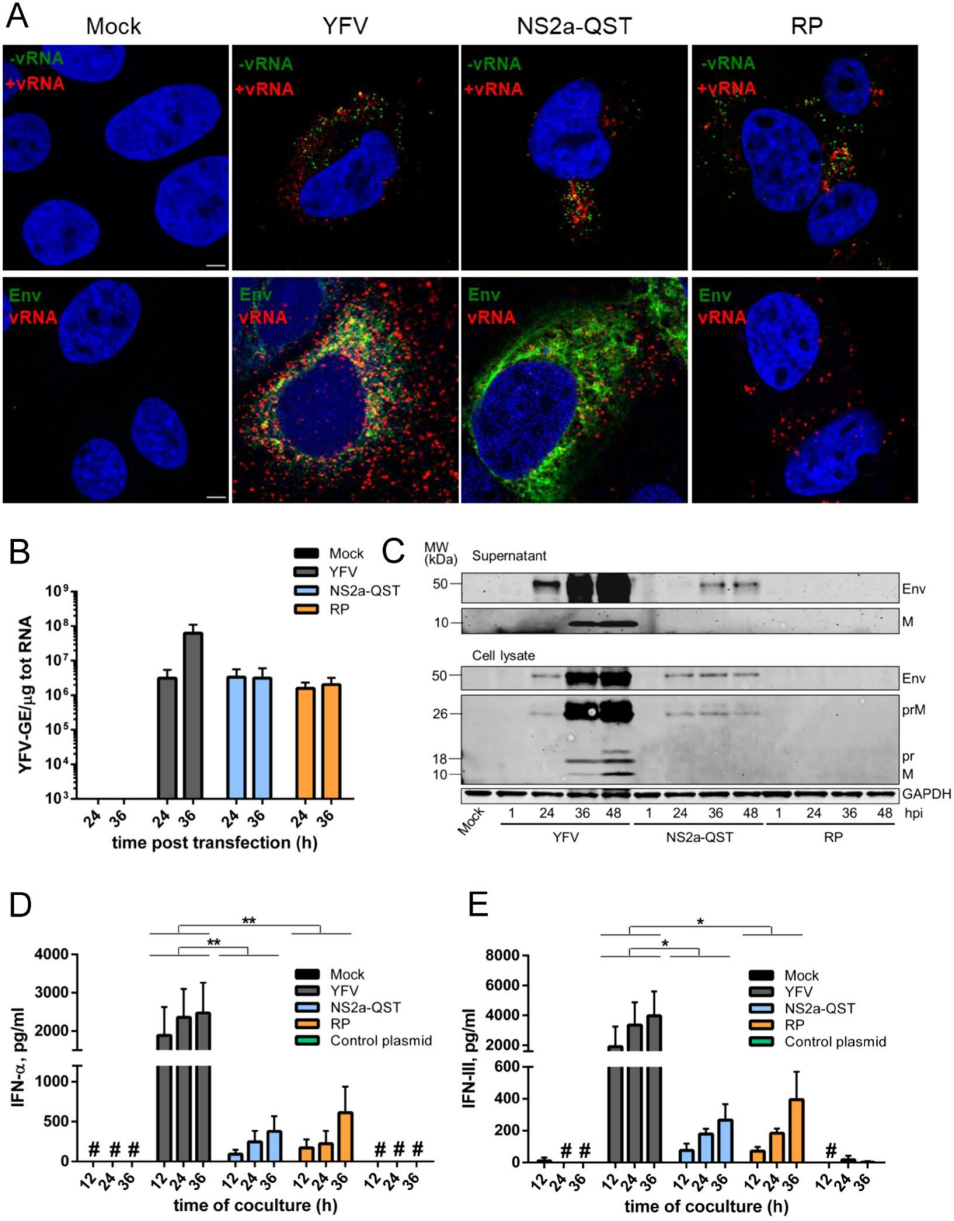
Packaging of YFV RNA into virions is not a requisite for pDCs stimulation by infected cells. Huh7.5 cells were untransfected (mock) or transfected with *in vitro*-produced RNAs corresponding to genome-length YFV, NS2a-QST YFV mutant or YFV subgenomic replicon (RP). (**A**) Cells were stained at 24 h post-transfection with NucBlue to visualize nuclei (blue) and processed for FISH using probes specific for (+) or (−) strand vRNA (red and green, respectively). Alternatively (lower panels), cells were stained with antibodies specific for viral Env protein (green) and with NucBlue to visualize nuclei (blue) and then processed for FISH using a probe specific for (+) strand vRNA (red). Images are representative of two independent experiments. Ten microscopic fields were analyzed. Scale bars are 5 μm. (**B**) The relative amounts of cell-associated viral RNA were determined by qPCR analysis at the indicated time post-transfection. Results are expressed as genome equivalents (GE) per μg of total cellular RNA (n = 4). (**C**) Whole-cell lysates and cell culture medium of the indicated cells were analyzed by immunoblotting at different time post-transfection using antibodies recognizing GAPDH or anti-YFV polyclonal ascites fluid. Marker protein sizes in kDa are reported on side. Representative blots of three independent experiments are shown. Full-scans blots are presented in Fig. S5. (**D,E**) Huh7.5 cells were mock-electroporated or electroporated as above for 24 h and then co-cultured with pDCs for the indicated time. Cell culture medium was analyzed by ELISA to determine the amounts of secreted IFN-α or IFN-III. ^#^Indicates values below the limit of detection of the assay. Data are means ± SEM of four independent experiments. Statistical analyses were performed using ANOVA tests.

To investigate further viral requirement for pDC stimulation, we took advantage of a previously characterized YFV mutant, YFV-NS2a-QST, which carries a single mutation in the NS2a protein, blocking the production of infectious particles without affecting viral replication^32^. We also included in our analysis a YFV subgenomic replicon (RP), which expresses uniquely NS proteins and is therefore unable to produce viral particles^33^. FISH analysis using probes recognizing either (+) and (−) strands of the viral genome revealed that Huh7.5 cells transfected with YFV full-length RNA, the NS2a mutant RNA or the subgenomic RNA were positive for both (+) and (−) viral RNAs (Fig. 7A, top panels). Since viral (−) strands are viral replication intermediates that are not packaged into virions, these data indicate that the NS2a mutant and the subgenomic RNA are replication-competent in Huh7.5 cells, like the full-length genome, as previously reported^32,33^. As expected, the viral Env protein was detected by immunostaining in cells transfected with full-length and NS2a-QST RNAs, but not in cells transfected with the subgenomic replicon (Fig. 7A, bottom panels). Analysis of viral copy numbers by RT-qPCR of Huh7.5 cells transfected with the different versions of the viral RNAs revealed that the NS2a mutant genome and the subgenomic replicon produced similar levels of viral RNA than the full-length genome at 24 hour post-transfection (Fig. 7B). The number of genomes increased of around 2 log between 24 and 36 hours in cells transfected with the full-length RNA whereas it remained stable over-time in cells expressing the mutant genome or the subgenomic replicon (Fig. 7B). This reflects the inability of the NS2a mutant and the replicon to spread within the culture^32,33^. Consistently, immunoblot analysis of both cell lysates and culture medium using anti-YFV ascites fluid, which binds Env and M proteins, showed that cells expressing full-length RNAs produced increasing amount of viral proteins and viral particles over-time (Fig. 7C). By contrast, the level of viral proteins produced in cells expressing the NS2a-QST genome did not increase over-time. As expected, no M or Env proteins were detected in cells transfected with the subgenomic RNA (Fig. 7C). Env proteins were detected in the supernatant of cells expressing the NS2a mutant, probably representing the released subviral Env-containing particles previously described^32^. Titration by plaque-assay of infectious particles released from transfected cells confirmed that cells harboring the NS2a mutant or the subgenomic RNAs did not produced infectious particle (Fig. S4A). Supernatants were also titrated by FFA, a variation of the plaque assay that employs immunostaining techniques, to rule out that viral particles, including Env-containing subviral particles, could have lost their ability to generate plaques by cell lysis and would therefore be undetected in the plaque assay. Viral titration by FFA confirmed that cells expressing the NS2a mutant or the replicon did not produce infectious viral particles, by contrast to cells expressing the full-length genome (Fig. S4B). Together, these data confirm that the mutant NS2a-QST did replicate as well as full-length viral RNA in Huh7.5 cells but did not release viral particles nor propagate within the cell culture. As expected, cells expressing the replicon did not produce viral particles.

We compared the ability of Huh7.5 cells expressing YFV full-length genome, the release-deficient NS2a mutant, the subgenomic replicon or a control plasmid to stimulate pDCs to produce IFN-α and IFN-III. Mock-transfected cells and cells expressing the control plasmid failed to stimulate pDCs (Fig. 7D,E). pDCs in contact with cells transfected with the NS2a mutant genome or the subgenomic replicon secreted IFN-α and IFN-III, albeit to a lesser extent than when in contact with cells expressing the full-length viral RNA (Fig. 7D,E). These data indicate that immunostimulatory viral RNAs can be transmitted to pDCs independently of capsid- and envelope-proteins.

## Discussion

Recent data generated in the context of infection with evolutionarily distant RNA viruses have revealed that sensing of virus-infected cells by pDCs was more effective than sensing of circulating cell-free viruses^10,11,13,34^. We report here that Huh7.5 cells infected with YFV were potent activators of pDCs. Contacts between YFV-infected cells and pDCs were a key feature for this activation, which parallels data obtained in the context of infection with unrelated RNA viruses^7,10,12–14,16^, or the closely-related DENV^6^. Such contacts likely favor the accumulation of RNA carriers within the intercellular space and facilitate their uptake by pDCs.

Two to three times more pDCs were connected to infected cells than non-infected ones, indicating that YFV infection stimulates and/or prolongs intercellular contacts. Infected cells might secrete yet-to-be identified chemo-attractants to recruit pDCs. Infection might also induce the expression of adhesion molecules at the surface of Huh7.5 cells, which could reinforce their contacts with pDCs. Once stimulated, pDCs might up-regulate the expression of adhesion molecules too, which would form long-lived contacts with infected cells.

Our data indicate that YFV RNA was transferred from cell-to-cell within carriers that were not neutralized by antibodies known to block viral entry and/or fusion. From this observation, we formulate several hypotheses about the nature of the vesicles that carry immunostimulatory viral RNA from Huh7.5 cells to pDCs. YFV RNA might be transported from cell-to-cell within single non-infectious viruses whose Env and M proteins escape neutralizing antibodies recognition. These could be prM-immature particles, which represent as much as 30 to 40% of the total number of viral particles produced by flavivirus-infected cells^35^. Consistently, modulating furin activities showed that cells producing immature YFV particles stimulated pDCs more potently than cells releasing mature infectious viruses. Therefore, as described in the context of DENV infection^6^, concentration of immature YFV particles at site of contact between infected cells and pDC participates in IFN production. Retention of fusion-incompetent immature particles in TLR7-positive compartment likely explains this phenomenon. Alternatively, YFV RNA could be carried from infected cells to pDCs within cluster of viruses protected by a biofilm-like shield, as reported for a retrovirus^36^ or by phosphatidylserine lipid-enriched vesicles, as described for enteroviruses^37^. These clustered viruses escape immune recognition. Our SEM analysis revealed the presence of viral clusters at the site of contact between YFV-infected cells and pDCs, indicating that this mode of transport could also be exploited by YFV. Finally, YFV RNA could be transported from infected cells to pDCs via exosomes, as described for HCV^7^.

Experiments performed with a release-deficient mutant and a YFV subgenomic replicon revealed that viral particle production from donor cells was not required for pDC response. As such, it is different from the mechanism of pDC stimulation by HIV-1- and DENV-infected cells, which rely on the presence of the viral envelope^6,13^. Based on these results, we hypothesize that a subset of viral RNAs produced by infected cells might traffic from Huh7.5 cells to pDCs without being released into the extracellular milieu and that this process contributes, at least in part, to pDC response. Capsid-free viral RNA might associate with cellular proteins to form viral ribonucleoprotein (vRNP) complexes and travel from one cell to another by utilizing existing cell-cell contacts, as described for the RNA of plant viruses, such as Tobacco mosaic virus^38^. Very recent data show that the genomic RNA of tick-borne encephalitis virus, another flavivirus, is transported within the dendrite of neurons independently of viral proteins^39^. The mechanisms by which such capsid-free viral RNA is transported between cells remain to be defined. The filopodia-like connections between Huh7.5 cells and pDCs could serve as intercellular channels to transfer viral RNA, either packaged within a viral particle or as an RNP complex. Such filopodia-mediated intercellular transport has been reported for human respiratory syncytial virus^40^.

Thus, viral RNAs produced by YFV-infected cells reach pDCs via at least two mechanisms: within immature particles and as capsid-free RNA. In conclusion, our study highlights the ability of pDCs to respond to a variety of viral RNA-laden carriers generated from infected cells.

## Materials and Methods

### Viruses, subgenomic replicon and infectious clones

The YFV vaccine strain (YF-17D-204 STAMARIL, Sanofi Pasteur, Lyon) was provided by the Institut Pasteur Medical Center. The stock was prepared on Vero cells, concentrated by polyethylene glycol 6000 precipitation and purified by centrifugation in a discontinuous gradient of sucrose. YFV infections were carried out at a multiplicity of infection (MOI) of 1, unless stated otherwise. The virus was titrated on Vero cells by plaque assay as described previously^9^. Alternatively, presence of viruses in cell culture media was evaluated by focus forming assay (FFA). For the latter assay, Vero cells were fixed at 5 days post infection with 4 PFA%, permeabilized with PBS containing 0,5% Triton X-100, saturated with PBS containing 0,05% Tween-20, 5% BSA (PBSTA) and incubated overnight with anti-Env MAb in PBSTA. Cells were then stained for 1 h with secondary anti-mouse antibodies conjugated to DyLight^TM^ 800 diluted in PBSTA. Plates were revealed using an Odyssey CLx infrared imaging system (LI-COR Bioscience). YFV was inactivated by exposure to UV light (4.75 J/cm^2^) for 10 min at a distance of 10 cm. The complete loss of detectable infectivity after UV exposure was confirmed by plaque assay. Sendai Virus (SeV)^22^ was used at 50 HAU/ml. The plasmids pACNR-FLYF17D-II and pACNR-FLYF17D-NS2a-QST, as well as the YFV subgenomic replicons were previously described^32,33,41^.

### Cell lines, antibodies and inhibitors

African green monkey kidney epithelial Vero cells (American Type Culture Collection; ATCC), HEK-293T cells (ATCC CRL-1573) over-expressing furin^6^, human hepatoma Huh7 (kindly provided by E. Meurs) and Huh7.5 cells^42^ (kindly provided by C.M. Rice) were maintained in Dulbecco’s modified Eagle’s medium (DMEM, Invitrogen), supplemented with 10% heat-inactivated fetal bovine serum (FBS), 1% MEM non-essential Amino Acid Solution and 1% penicillin-streptomycin (P/S, Sigma). The following antibodies were used: anti-GAPDH (Abcam), anti-IFN-α (Milteny, which recognizes all IFN-α subtypes except IFN-α2b), anti-actin Alexa-Fluor-488 conjugated (Life Technologies), anti-mouse 680 (LI-COR Bioscience), anti-rabbit 800 (Thermo Fisher Scientific), anti-mice Alexa-Fluor 647 (Life Technologies), anti-mice Alexa-Fluor 488 (Life Technologies), APC-conjugated anti-CD303 (BD Pharmingen) and anti-CD123, FITC-conjugated, or not (BD Pharmingen). Anti-YFV ascites fluid generating using the French Neurotropic Vaccine strain and Env MAb 4G2 hybridoma cells were kindly provided from P. Desprès. YFV-NS4b and YFV-prM were gifts from C.M. Rice^43^. IgG2a-isotype controls were from Abcam. The furin inhibitor Decanoyl-RVKR-CMK (Calbiochem) was used in Huh7.5 cells at the indicated concentrations.

### Cell viability assays

Cell viability was determined using the CellTiter-Glo® luminescent viability assay (Promega) according to the manufacturer’s recommendations. This assay is based on ATP quantification as indicator of metabolically active cells.

### Preparation of PBMCs, depletion and purification of pDCs

The blood donors, whose age ranged between 21 and 66 years, were randomly selected from a population of healthy male and female volunteers donating blood at the ‘Etablissement Français du Sang’ (EFS), within the framework of an agreement with Institut Pasteur. Experimental procedures with human blood have been approved by EFS Ethical Committees for human research. All samples were collected in accordance with EU standards and national laws. Informed consent was obtained from all donors. Blood samples were negative for HCV, Hepatitis B virus, HIV-1, human T-lymphotropic virus and Cytomegalovirus. They were not screened for reactivity to YFV. PBMCs were purified from a unique donor using Ficoll-Plaque Plus solution (GE Healthcare). They were re-suspended in RPMI-1640 medium supplemented with 10% FBS, 1% P/S or in PBS depending on the experiment. pDCs were isolated using the pDC Isolation Kit II (Miltenyi) through depletion of non-pDCs. Briefly, freshly prepared PBMCs were incubated with a cocktail of non-pDC biotin-conjugated antibodies and then with non-pDC MicroBeads. Cells were then isolated using an AutoMACS Pro Separator (Miltenyi). pDC-depleted PBMCs were also collected. pDCs purity and pDC-depletion efficiency were determined by flow cytometry using antibodies against CD303 and CD123. pDCs were maintained in RPMI-1640 medium supplemented with 10% FBS and 1% P/S.

### pDC stimulation and TLR7 inhibition

pDCs were stimulated either with Sendai virus, cell-free YFV or crude supernatant of YFV-infected Huh7.5 cells. For co-culture experiments, Huh7.5 cells were seeded at 3.10^5^ cells per condition and mock-infected or infected with YFV. Sixteen hour post-infection, around 10^5^ pDCs, 3.10^5^ PBMCs or pDC-depleted PBMCs were co-cultured with Huh7.5 cells for further 24 hours. For transwell assays, Huh7.5 cells were cultured in the lower compartment and pDCs in the upper one (membrane pore size of 0.4 μm, Coster Corning Corp.). For the inhibition of TLR7, pDCs were co-cultured as described above and incubated for 24 hours with the TLR7 inhibitor ODN A151 (InvivoGen) or ODN control (Eurofins Genomics) at a final concentration of 5 μM.

### *In vitro* transcription and transfection of Huh7.5 cells

Plasmids pACNR-FLYF17D-II, pACNRFLYF17D-NS2a-QST and YF-R.luc2A-RP were linearized with the restriction enzyme *Xho*I (NEB), recovered by phenol-chloroform extraction and ethanol precipitation. The DNA was used as a template for SP6 *in vitro* transcription using the mMESSAGE mMACHINE SP6 Transcription Kit (Ambion). The *in vitro*-transcribed RNAs were purified by phenol-chloroform extraction and ethanol precipitation and re-suspended in RNase-free water. The control RNA was generated from the linearized pTRI-Xef control template DNA (TRIPLEscript plasmid containing the 1.85-kb Xenopus elongation factor-1). Sub-confluent Huh7.5 cells (8.10^6^ per condition) were trypsinized, washed and re-suspended in cold PBS. Electroporation of the RNAs was carried out in 2-mm GenePulser cuvettes (BioRad) at 270 volts and 500 μF using a GenePulser system (Bio-Rad). After electroporation cells were allowed to recover at RT for 5 min, re-suspended in complete growth media and plated at 3.10^5^ cells per condition. Media was replaced after 2 hours of incubation at 37 °C.

### RNA extraction and RT-qPCR analysis

RNAs were extracted from cell-lysates or cell-culture supernatants using the NucleoSpin RNA II Kit (Macherey-Nagel) following the manufacturer’s protocol and were eluted in nuclease-free water. First-strand complementary DNA synthesis was performed with the RevertAid H Minus M-MuLV Reverse Transcriptase (Thermo Fisher Scientific). Quantitative real-time PCR was performed on a real-time PCR system (QuantStudio 6 Flex, Applied Biosystems) with SYBR Green PCR Master Mix (Life Technologies). Viral genome equivalents concentrations (GE/ml) were determined by extrapolation from a standard curve generated from serial dilutions of the plasmid encoding the YF-R.luc2A-RP^33^. YFV-NS3 primers were used for the RT-qPCR (sense 5′-AGGTCCAGTTGATCGCGGC and antisense 5′-GAGCGACAGCCCCGATTTCT).

### ELISA

The amount of IFNs in cell-culture supernatants was quantified by Enzyme-Linked Immunosorbent Assay (ELISA) using IFN-α pan (Mabtech) and IFN-III (PBL Interferon Source) kits, according to manufacturer’s instructions. The IFN-α pan kit detected IFN-α subtypes 1, 2, 4, 5, 6, 7, 8, 10, 13, 14, 16 and 17. The IFN-III kit detected IFNλ1 (IL-29), IFNλ2 (IL-28A) and IFNλ3 (IL-28B). The detection limits were 31 pg/ml for IFN-α and 63 pg/ml for IFN-III.

### Neutralization assays

Huh7.5 cells were seeded at 3.10^5^ cells per condition and infected for 16 hours at a MOI of 0,2. Cells were extensively washed and incubated for 24 hours with IgG2a-isotype control (20 μg/ml), with anti-Env MAb (20 μg/ml) or with anti-YFV ascites fluid (dilution ranging from 1:50 to 1:1000). After 24 hours, the supernatants were collected and analyzed by plaque assays. Alternatively, cells infected for 16 hours were incubated for 1 hour at 37 °C with the different antibodies, prior of adding 5.10^5^ PBMCs treated with FcR blocking antibodies (Miltenyi). After 24 hours, cell culture media were analyzed by plaque assays, RT-qPCR or ELISA.

### Western blot analysis

Cells were lysed with RIPA buffer containing a protease inhibitor cocktail (Roche Applied Science, ref 99727). Cell lysates were normalized for protein content, boiled in NuPAGE LDS sample buffer (Thermo Fisher Scientific) in non-reducing conditions and the proteins were separated by SDS-PAGE (NuPAGE 4–12% Bis-Tris Gel, Life Technologies). Cell supernatants were pelleted through 20% sucrose in TNE (20 mM TrisHCl pH 8.0, 150 mM NaCl, 2 mM EDTA) before separation by SDS-PAGE. Separated proteins were transferred to a nitrocellulose membrane (Bio-Rad). After blocking with PBS-Tween-20 0,1% (PBST) containing 5% milk for 1 hour at RT, the membrane was incubated overnight at 4 °C with primary antibody diluted in blocking buffer. Finally, the membrane was incubated for 1 hour at RT with secondary antibodies diluted in blocking buffer, washed, and scanned using an Odyssey CLx infrared imaging system (LI-COR Bioscience).

### Immunofluorescence, fluorescence *in situ* hybridization (FISH) and confocal analysis

pDCs were stained with the Cell Tracker Deep Red Dye (Thermo Fisher Scientific) for 15 min at 37 °C. Labeled pDCs were co-cultured with previously seeded and infected Huh7.5 cells, in glass bottom 96-well plates (Eppendorf). After fixation with PFA 4% for 30 min at RT, cells were permeabilized with PBS, 0,5% Triton X-100 for 10 min at RT, blocked with PBSTA for 30 min and stained with anti-Env MAb in PBSTA for 1 hour. Cells were then stained with secondary antibodies Alexa-Fluor 488 (Life Technologies) in PBSTA for 30 min. For the IFN-α staining, cells were treated with Brefeldin A (BFA, Sigma) at 5 μg/ml during the last 3 hours of co-culture. The anti-IFN-α antibody (Miltenyi), which recognizes all IFN-α subtypes, at the exception of IFN-α2b, was used at 1:50 dilution in BD perm/wash buffer 1X (BD Pharmingen). After immunostaining, the (+) and (−) RNA strands of YFV were detected following the manufacturer’s protocol (ViewRNA ISH Cells Assays) using probe sets designed by Affymetrix. The Alexa-Fluor 546-conjugated (+)-strand probe set targets a region between position nt 4567 and 5539 of the YFV genome. Alexa-Fluor 488-conjugated (−)-strand probe set recognizes a region between nt position 3238 and 4110 of the viral genome. Nuclei were stained with NucBlue (Thermo Fisher Scientific) for 15 min at RT. Z-sections across cells at 0.23-μm increments were acquired with a Zeiss LSM 720 laser scanning confocal microscope equipped with a X63 objective. When indicated, images were analyzed with the ICY software (icy.bioimageanalysis.org) using the Spot Detector plugin. A written script was used to defined regions of interest representing the whole volume of individual pDCs. Deconvolution and 3D-reconstruction of individual cells or pDC/Huh7.5 cells co-culture were performed using Huygens Essentials software (Scientific Volume, Imaging BV) and a volume-rendering algorithm.

### Flow cytometry and RNA flow assays

Huh7.5 cells were co-cultured with pre-labeled pDCs (Cell Proliferation Dye eFluor 450, eBioscience) and infected with YFV for 24 hours. For protein detection, cells were fixed with BD cytofix/cytoperm kit (BD Pharmingen). Primary and secondary antibodies were incubated for 1 hour at 4 °C. Data were collected with an Attune NxT Flow Cytometer (Thermo Fisher Scientific). RNA detection by flow cytometry was performed according to the manufacturer’s instructions (PrimeFlow RNA Assay, Affymetrix eBioscience). After fixation and permeabilization, cells were incubated for 2 hours at 40 °C with customized YFV-17D (Alexa-Fluor 647) and hIFN-α (Alexa-Fluor 488) probe sets that recognize IFN-α subtypes 1 and 2 (Affymetrix, eBioscience). Data were collected with a Fortessa Flow Cytometer (BD Biosciences). All data were analyzed with FlowJo software.

### Scanning Electron Microscopy

pDCs co-cultured with Huh7.5 cells were fixed with 2.5% Glutaraldehyde in 1X PHEM buffer (PIPES, HEPES, EGTA and MgCl_2_), supplemented with 0,1 M sucrose and 0,05% MgCl_2_ for 30 min at RT. Preparations were rinsed with 2X PHEM buffer and then fixed with 1% Osmium tetroxide for 1 hour, dehydrated in increasing concentration of ethanol (from 25, 50, 75, 95 and 100%) and then further dehydrated by the method of critical point drying. Finally, the samples were subjected to sputter-coating with 10 nm Au/Pt (gold/platinum). For immunogold labeling, cells were fixed with PFA 4%, quenched with 0,1 M glycine in PBS and blocked with PBS-5% BSA for 1 hour. Cells were stained using anti-Env MAb or CD123 in PBS-5% BSA for 1 hour followed by anti-mouse IgG2a antibody in PBS-5% BSA for 15 min and protein A-gold (20 nm) conjugated in PBS-5% BSA. Samples were processed as before with a final passage of metallization of 3 nm carbon. Samples were imaged using a JEOL JSM-6700F SEM.

### Statistical analysis

Data were analyzed using GraphPad Prism 7. Statistical analyses were performed with Student’s unpaired or paired two-tailed t test, as indicated. When comparing more than 2 experimental conditions, one-way analysis of variance (ANOVA) was performed. Data are presented as means ± SD or SEM of at least three independent experiments. Statistically significant differences are indicated as follows: *p < 0.05, **p < 0.01, and ***p < 0.001; ns, not significant.

## Electronic supplementary material

Supplementary Information
