# Supplementary material for: Immature particles and capsid-free viral RNA produced by Yellow fever virus-infected cells stimulate plasmacytoid dendritic cells to secrete interferons
Source: Sci Rep. 2018 Jul 18;8:10889. doi: 10.1038/s41598-018-29235-7 (PMC6052170; doi:10.1038/s41598-018-29235-7)
Supplement: Supplementary file 1 — Supplementary Information [file 41598_2018_29235_MOESM1_ESM.pdf]

Supplementary Information (**SI**) for

Immature particles and capsid-free viral RNA produced by Yellow fever virus-infected cells stimulate plasmacytoid dendritic cells to secrete interferons

Laura Sinigaglia[1], Ségolène Gracias[1], Elodie Décembre[2], Matthieu Fritz[3], Daniela Bruni[1], Nikaïa Smith[4], Jean-Philippe Herbeuval[4], Annette Martin[3], Marlène Dreux[2], Frédéric Tangy[1], Nolwenn Jouvenet[1*]

[1]Viral Genomics and Vaccination Unit, UMR3569 CNRS, Institut Pasteur, Paris, France. [2]CIRI, Inserm U1111, CNRS UMR5308, École Normale Supérieure de Lyon, Université Claude Bernard Lyon 1, Lyon, France. [3]Molecular Genetics of RNA Viruses Unit, UMR3569 CNRS, Institut Pasteur, Paris, France. [4]Chemistry & Biology, Modeling & Immunology for Therapy, UMR8601 CNRS, Université Paris Descartes, Paris, France.

[*]Address correspondence to NJ (email: nolwenn.jouvenet@pasteur.fr)

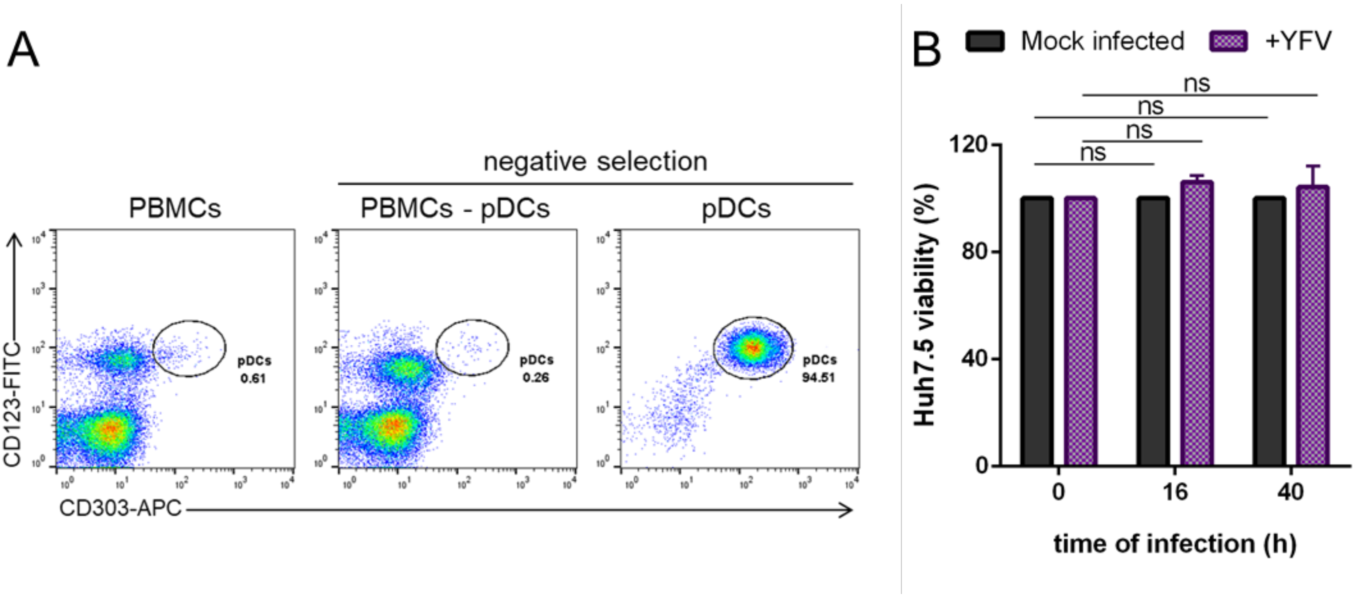

Figure S1. **(A)** Analysis of the depletion or isolation of pDCs from human PBMCs. Analysis of CD123 and CD303 (BCDA-2) surface expression of PBMCs, pDC-depleted PBMCs (PBMCs -pDCs) or purified pDCs by flow cytometry. The purity of isolated preparations of pDCs was always >94%. **(B)** Analysis of Huh7.5 cells viability upon YFV infection. Huh7.5 cells were left uninfected or infected with YFV for the indicated times. Cell viability was determined using a luminescent-based assay. Luciferase activities (± SD) were expressed as percentage relative to non-infected cells (n=3). Statistical analysis was performed using an ANOVA test.

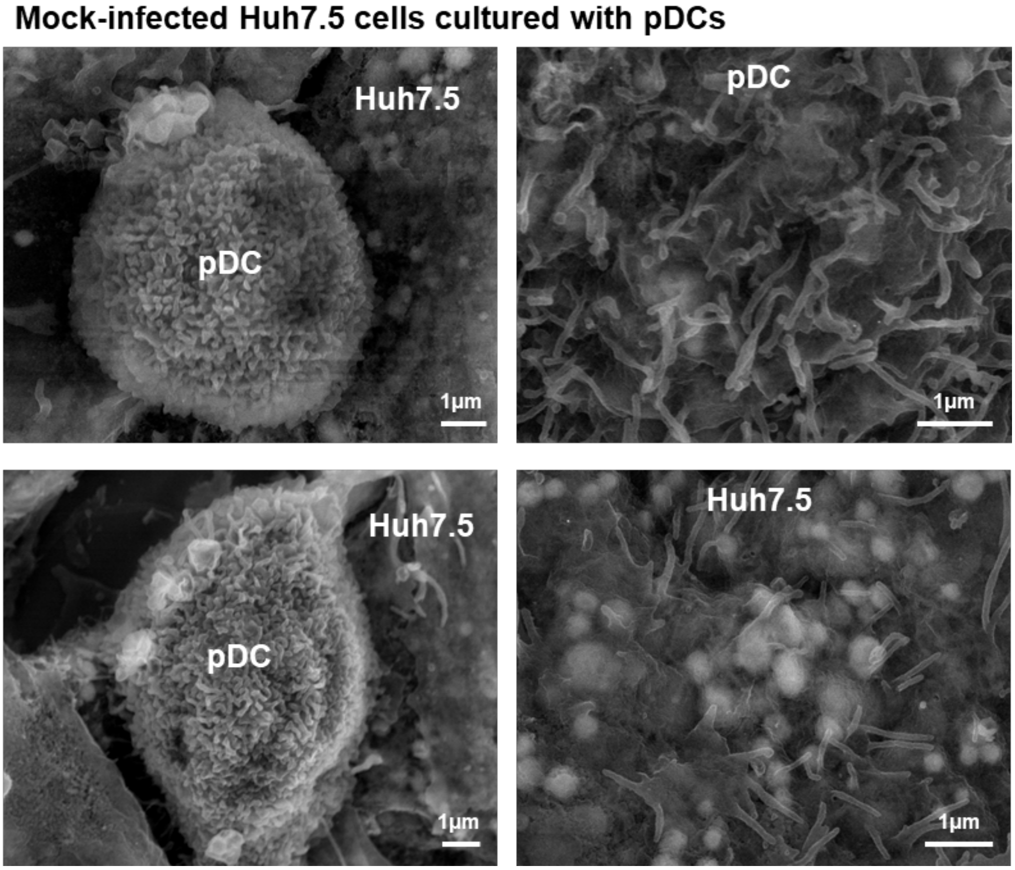

Figure S2. No Env signal was detected by SEM analysis of non-infected cell culture. pDCs were co-cultured with mock-infected Huh7.5 cells for 24 hours, stained with anti-Env antibodies coupled to gold particles and process for SEM analysis. Scale bars are as indicated.

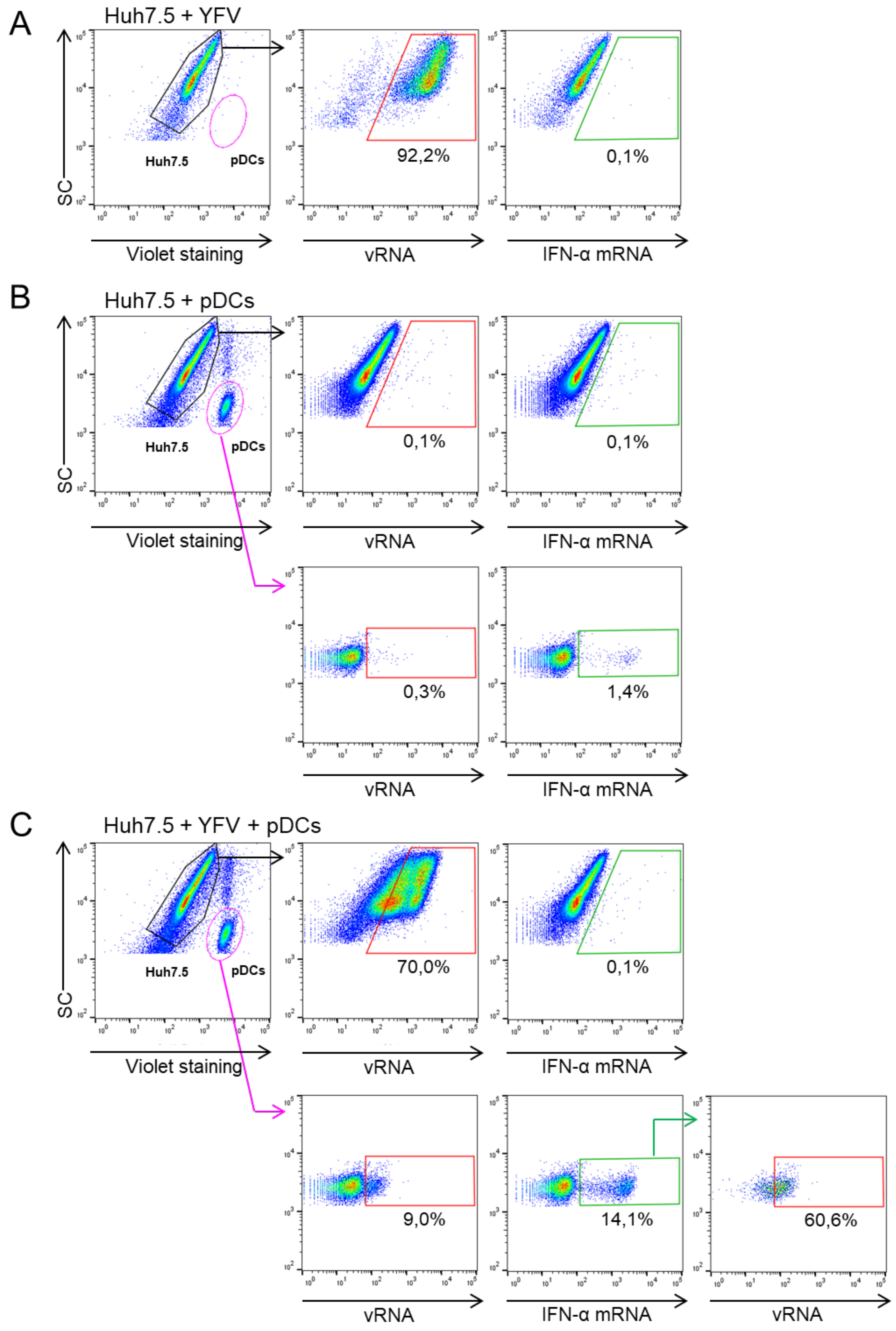

Figure S3. Analysis by Flow-FISH of the proportion of stimulated pDCs within the whole population. **(A)** Huh7.5 cells were infected with YFV at a MOI of 1 for 40 h. **(B)** Non-infected Huh7.5 cells were were co-cultured with eFluor 450-labeled pDCs for 24 h. **(C)** Huh7.5 cells were infected with YFV at a MOI of 1 for 16 h and then were co-cultured with eFluor 450-labeled pDCs for a further 24 h. (**A-C**) Cells were incubated with probes that target vRNA and IFN-α1/2 mRNA, respectively, and then analyzed by flow cytometry. Quantifications representative of three independent experiments are shown in figure 4C.

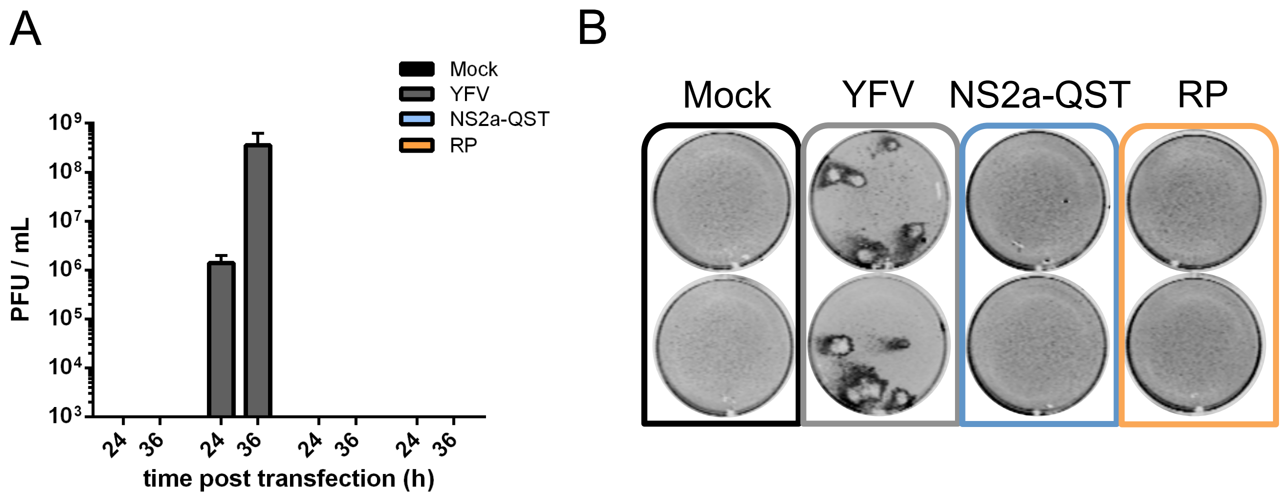

Figure S4. Cells expressing the YFV-NS2a-QST mutant or the YFV replicon do not release infectious particles. (**A-B**) Huh7.5 cells were mock-electroporated or electroporated with RNA generated from a plasmid expressing wild-type YFV, a YFV mutant carrying a mutation in the NS2a protein (NS2a-QST) or a YFV replicon (RP). (**A**) At 24h post-electroporation, presence of infectious viruses in cell culture media was assessed by plaque assay on Vero cells (expressed as PFU/ml). Data are means ± SD of three independent experiments. (**B**) Alternatively, the presence of infectious viruses in cell culture media was evaluated by focus forming assay (FFA) on Vero cells using anti-Env 4G2 antibodies.

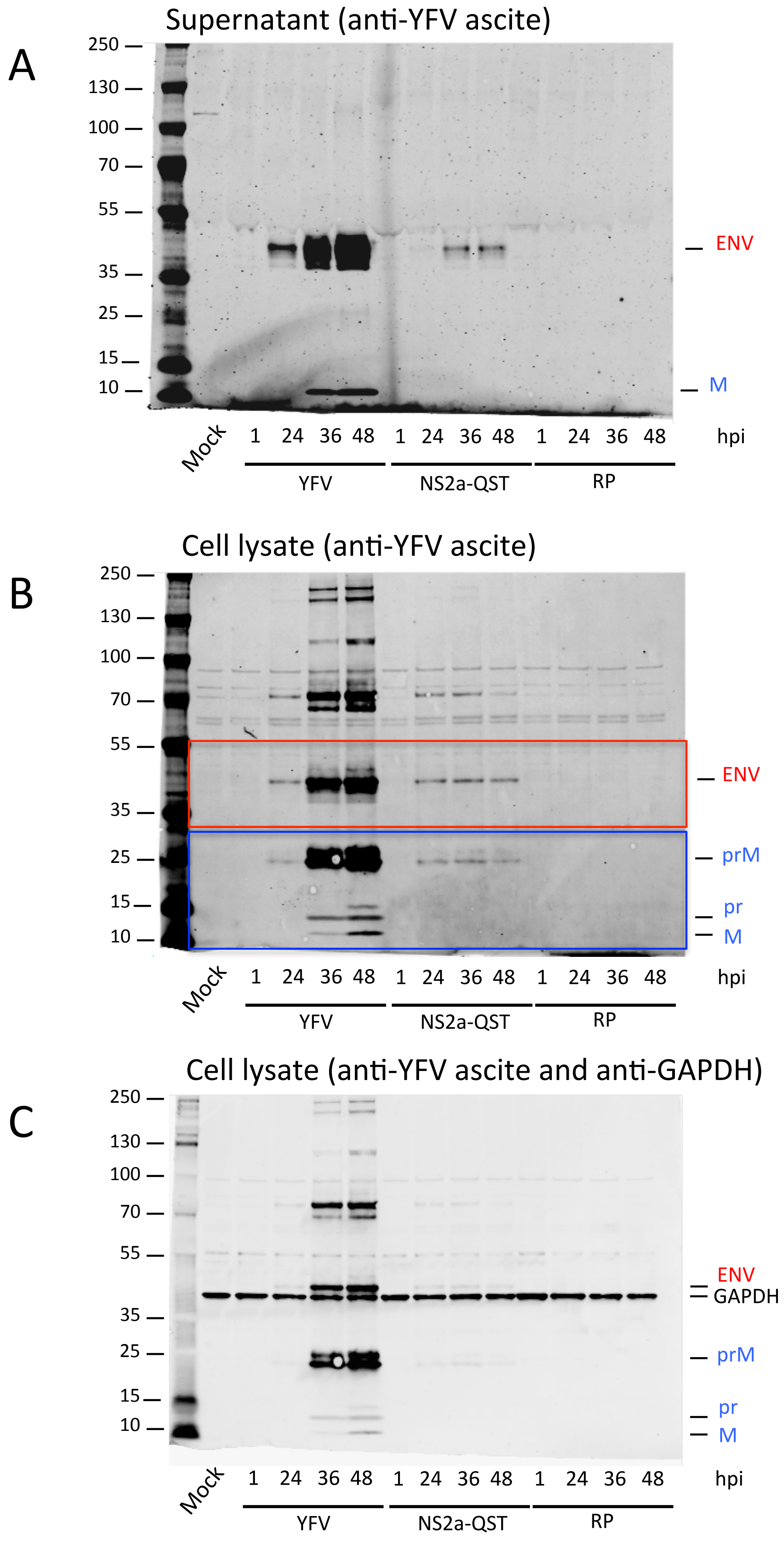

Figure S5. Full scans of western-blots shown in figure 8C. The whole-cell lysates samples in panel B were probed with anti-YFV polyclonal ascites fluid. The blue and red boxes correspond to the images shown in figure 8C. The same membrane was later analyzed using antibodies recognizing GAPDH (panel C). Marker protein sizes in kDa are reported on side.
